# Testing the Role of Waste Management and Environmental Quality on Health Indicators Using Structural Equation Modeling in Pakistan

**DOI:** 10.3390/ijerph18084193

**Published:** 2021-04-15

**Authors:** Tanzila Akmal, Faisal Jamil

**Affiliations:** School of Social Sciences and Humanities, National University of Sciences and Technology (NUST), Islamabad 44000, Pakistan; Faisal.jamil@s3h.nust.edu.pk

**Keywords:** sustainable solid waste management, health status, structural equation modeling

## Abstract

Improper management of municipal waste has become a growing concern globally due to its impact on the environment, health, and overall living conditions of households in cities. Waste production has increased because households do not adopt waste management practices that ensure sustainability. Previous studies on household waste management often considered socio-economic aspects and overlooked the environmental and behavioral factors influencing the disposal practices and health status. This study adopted four constructs, defensive attitude, environmental knowledge, environmental quality, and waste disposal, by employing a structural equation modeling approach to explore research objectives. Data from 849 households of the Islamabad-Rawalpindi metropolitan was collected by using a multi-stage sampling technique. The structural model results showed that the two constructs, environmental knowledge and defensive behavior, positively affect household health status. The most significant health-related considerations are waste disposal and environmental quality, both of which negatively impact health status and do not support our hypothesis. The results provide valuable perspectives to enable households to engage actively in waste management activities. The findings indicate that understanding the intentions of household health status drivers can assist policymakers and agencies in promoting an efficient and successful community programmes related to sustainable solid waste management by allowing them to foster how the desired behavior can be achieved.

## 1. Introduction

Municipal solid waste (MSW) is an important economic and environmental issue around the globe. MSW management is already a critical concern for municipal authorities, especially in emerging economies, due to the exponential increase in waste generation parallel with population growth, increasing living standards, urbanization, and rapid development [1,2]. In parallel, MSW management authorities lack infrastructure and the capacity to safely collect and dispose of waste to meet the growing demand. Rural-to-urban migration in emerging economies has resulted in unplanned urban settlements, which put tremendous pressure on municipality authorities. As a result, coping with household solid waste has become a big stumbling block for urban growth. Nevertheless, there is a gap between the demand and supply of these services in terms of quality and efficiency [3,4].

The MSW problem has become an important challenge to sustainable development in developing countries [5]. The lack of resources coupled with municipalities’ weak institutional capacity to comply with existing solid waste management structure, insufficient facilities for collection, transport, treatment and disposal of waste, limited technical competence and low level of public knowledge have made solid waste management difficult for local authorities [3,6]. Improper waste management leads to waste spreading along the roadsides, drainage, and haphazard dumping, all of which pose a serious risk to the environment and health [7] and urban flooding and waterlogging [8].

Open dumping and waste burning have been related to major public health hazards and contamination sources, resulting in the release of harmful dioxins and other toxic substances. At very low doses, these compounds cause a surprising range of harmful effects in humans. Adaptation of defensive behavior is a cognitive process of individuals, including people’s value and belief systems, attitudes and perceptions, personalities, motivations, aspirations, and community, to reduce the negative effects of excessive waste disposal. These cognitive factors drive household decisions about the hazardous impact of waste on human health and the environment and the essence of their reaction to negative impacts have prompted environmental psychologists to pay more attention to psychological aspects of climate change adaptation [9,10].

Pakistan’s population has been rising at a rate of 2.4% per year since 1998, reaching a peak of 207.7 million in 2017, which corresponds to the sixth most populous country. Islamabad is the capital and tenth-largest city with a 1.019 million population and Rawalpindi is the 4th largest city with 2.09 million inhabitants [11]. The average waste generation rate varies from 1.896 kg/house/day to 4.29 kg/house/day. Although the waste collection system is inadequate, the average waste collection rate in Pakistan’s public sector is 50% [12]. Open dumping is the most common practice, and dumping sites are often set on fire to reduce the amount of waste that accumulates, which has adverse effects on health and the environment. Public health and societal life are affected by health hazards, pest proliferation, and the spread of diseases. Municipalities fail to manage solid waste due to financial constraints and the careless behavior of the inhabitants. Solid waste has negative impacts on the environment, including air, soil, water contamination, climate change, and devastating effects on the flora and fauna [13,14].

The contribution of this study covers three aspects. First, to the authors’ knowledge, there was no inclusive research in Pakistan on household environmental and defensive behaviors in relation to waste disposal and studies that have generally investigated household’s defensive behaviors have been limited in Pakistan [15], although there has been some work on the environmental quality and adaptation for the poor sewage system in Pakistan [15,16,17,18,19]. Second, the study is of great worth in monitoring, controlling and humanizing local peoples’ waste management behavior. Specifically, the current study analyzes the impact of different socio-psychological variables (environmental quality, environmental knowledge, and defensive behavior) on health status that has received little attention. Accordingly, this study focused on the metropolitan area of Rawalpindi-Islamabad, Pakistan in order to gain a better understanding of the social economic and environmental factors that influence health. Third, our study also provides viable policy options for mitigating the health hazards of waste pollution and poor environmental quality within the Asian region since we share a common culture, so question is therefore also relevant to other countries in the Asian region.

## 2. Theoretical Model

### Inter-Relationships between Constructs

The need for environmental conservation in society has gradually increased. Human activities and anthropogenic impacts have a substantial adverse environmental effect [19,20]. In this regard households have different solid waste management preferences. In general, individuals make their choices based on the assumptions of rationality and self-interest.

**Figure 1:**
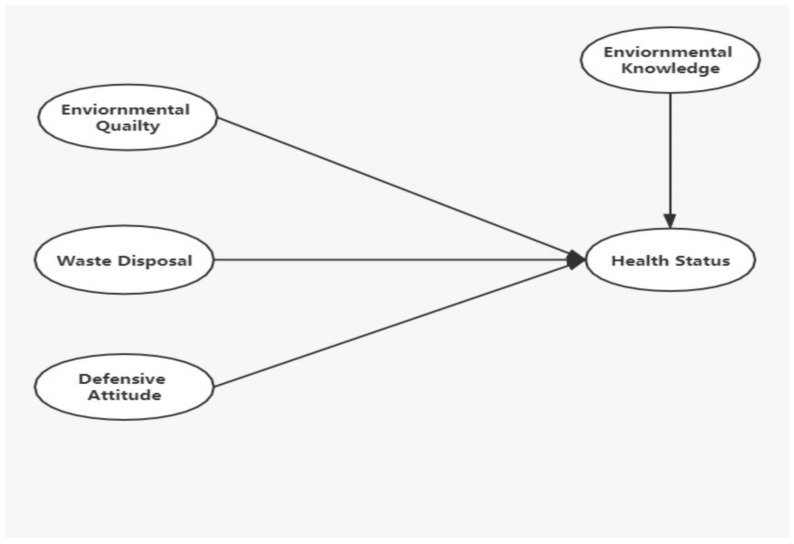
Structural model of the hypotheses

Several studies have examined the role of key socio-economic and demographic variables such as age [21], income, educational attainment [22] and health status. Waste is the product of human and economic activity, and it is determined by person, ecosystem, and community behavior. Solid waste is a significant environmental problem that jeopardizes long-term environmental sustainability [23]. Therefore, the following hypotheses are put forth based on theoretical framework (see Figure 1):

**Hypotheses** **1** **(H1).** *Waste disposal is positively and significantly associated with health status.*

The researchers have made significant efforts to relate improper solid waste management to health issues such as respiratory disorders, vector disease, aesthetic damage, drain blockage, water and soil contamination [2,24]. Environmental deterioration through waste pollution, air and water quality contribute significantly to the proliferation of diseases [25,26]. The consumption and waste disposal habits of households have a direct effect on the environment [27,28,29].

**Hypotheses** **2** **(H2).** *Environmental quality is positively and significantly associated with health status.*

The social and consumption behavior of households are imperative factors that contribute to waste generation and disposal. The social and consumption behavior of households depends on environmental knowledge. As a result, environmental awareness leads to defensive behavior, which is needed to avoid the harmful effects of solid waste [30,31]. As a result, households are encouraged to participate in hygiene waste management programs to reduce the negative impact on public health and the environment. Therefore, health and environment should be understood as two essential inseparable development aspects that cannot be sustained as though they operate in a vacuum [32,33].

**Hypotheses** **3** **(H3).** *Defensive behavior is positively and significantly associated with health status.*

Household defensive behavior is motivated by awareness of potentially harmful effects, as well as time and resources. Previous research [34,35,36] looked at several incidents in various parts of the world. According to these reports, households that have been exposed to certain catastrophe circumstances are more risk-averse. Individuals who are aware of then issue are more likely to respond and engage in risk-reduction practices. Based on the above literature, we develop the following hypotheses.

**Hypotheses** **4** **(H4).** *Environmental knowledge is positively and significantly associated with health status.*

## 3. Research Methods

### 3.1. Data Collection

To achieve the study’s objectives, data on household waste management practices environmental quality, environmental knowledge, defensive behavior and health status were gathered from 849 respondents. For selecting the sample size and study area, several factors have been taken into consideration such as the socio-economic and demographic characteristics of selected households for survey. A “multi-stage systematic technique” was used to choose the study area and household sample size.

So far, Pakistan does not have an institutional review board or national ethical guidelines for social science studies. Therefore, the study adhered to existing research ethics principles such as obtaining verbal consent to participate in research, safeguarding personal data, informal privacy, and allowing participants to withdraw their consent if they so wished at any point. In addition, no personal information was used in this analysis. Participants, who provided information related to solid waste generation and related information, were used in this research.

A questionnaire has been finalized after conducting pre-testing in the field. Pre-testing helped us to construct a better contextualize and revised questionnaire. A five-point Likert scale 1 = strongly disagree; 2 = disagree; 3 = neutrality; 4 = agree; 5 = strongly agree, was used to evaluate each question in the questionnaire. We have designed six questions to measure households’ waste disposal behavior, five questions for environmental quality, six questions on environmental knowledge, six questions on defensive behavior. Finally, we have designed four questions related to household health. Precise questions are shown in Table 1. Primarily data was input into the Statistical Package for the Social Sciences (SPSS) software (IBM, Armank, NY, USA) to generate descriptive statistics and their frequency and correlation test. Finally, we conducted a structural equation analysis through Analysis of Moment Structures (AMOS 20). Social-economic information of respondents is given in Appendix A (see Table A1).

### 3.2. Measurement Model (MM)

In this analysis, the structural equation modeling (SEM) method is used to evaluate the data using latent constructs in this study. To test our model, we used the Anderson and Gerbing’s [35] two-step approach. The first step was to establish a satisfactory measurement model (MM) using confirmatory factor analysis (CFA). The MM included latent constructs for environmental awareness, environmental quality, waste disposal, safety, and health status. Confirmatory factor analysis was used to determine the reliability of constructs. In additional, convergent and discriminant validity is used to evaluate construct validity. The magnitude, direction, and statistical significance of each latent construct’s standardized factor loadings were checked for convergent validity. Additionally, using the average variance extracted (AVE) and the building reliability, convergent validity was investigated. A MM is valid when a minimum AVE level is higher than 0.5, and when the minimum value of CR is higher than 0.7 [36].

Maximum likelihood estimation in structural equation modeling assumes multivariate normality. We looked at the univariate distributions for each component because assessing all aspects of multivariate normality is difficult. This method can be used to determine multivariate normality [37]. Multivariate collinearity was calculated by running multiple regressions, each with a different item as the dependent variable and the rest of the items as the independent variables, and then analyzing the tolerance and variance inflation factor (VIF) for each regression [37]. We measured each statement’s communality extraction to check the reliability and validity of each construct scores above 0.5., which showed that each factor is independent [37,38].

**Figure 2:**
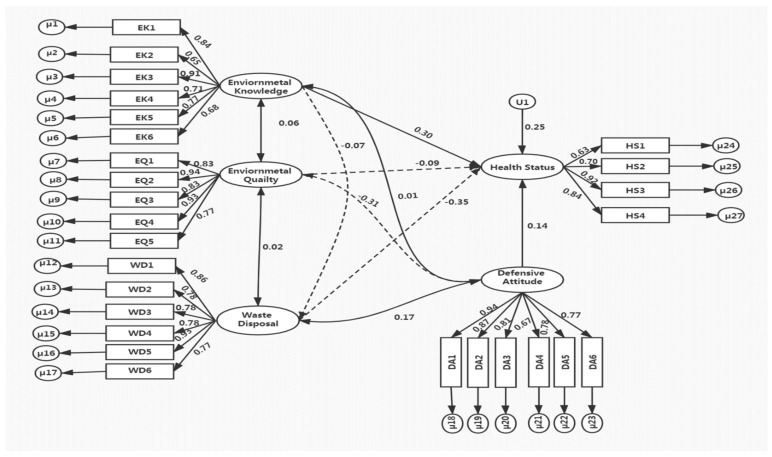
Structural equations modeling and path coefficients between variables.

After we attained a rational measurement model, the structure model was calculated to test the health status hypotheses. Structural modeling is used to predict relationships between households’ cognitions constructs (environmental knowledge, environmental quality, waste disposal, defensive behavior) and their health status. The SM is shown in Figure 2.

## 4. Results

The first step is to test the reliability test of survey data. There are two generic measures for reliability: Cronbach’s α and composite reliability [39]. The Cronbach’s α value is used to check the reliability of the data. Data is consistent when Cronbach’s α lies between 0.60 and 0.70; the data set used in analysis is highly reliable when the value is between 0.70 and 0.80 and cut off scores for composite reliability is between 0.6 and 0.7 [40]. SPSS 23.0 was used to check the internal reliability of five constructs (environmental knowledge, environmental quality, waste disposal, defensive behavior and health status). The results of Cronbach’s α values for five latent variables; waste disposal, defensive behavior environmental knowledge, environmental quality, and health status is 0.92, 0.92, 0.89, 0.93, and 0.85 respectively revealed good internal consistency.

A confirmatory factor analysis was applied to check the properties of the measurement scale [41]. The conventional rules of thumb [37] are followed for goodness-of-fit indices of the confirmatory factor analysis. Reliability tests try to find the stability and consistency of measuring instruments. Confirmatory factor analysis shows goodness-of-fit and specific indices for the empirical data such as chi-square standardized by degrees of freedom (λ/df) is shown in Table 2. It should be less than five [42], in our study it is 3.71. The NFI, and CFI should exceed 0.9 and RMSEA should be less than 0.10 [43]. Here, goodness of fits was as follows; NFI = 0.931, CFI = 0.948, and RMSEA = 0.057. Thus, results showed that the model could be accepted for empirical analysis with good convergent indices and goodness of fit [37,38,39,40,41,42,43,44]. Results of correlation test are given in Appendix A (please see Table A2).

## 5. Discussion

Results show that SEM is an appropriate methodology for explaining the behavior of the metropolitan Islamabad-Rawalpindi area towards waste management. The configuration of the MM and SM was appropriate. In four-specified MM, the latent constructs waste disposal, environmental quality, environmental knowledge, defensive behavior was reliably described by the measurable items. All the standard coefficients of estimated SEM revealed that path analysis (Figure 2) specified the relationships’ strength among all variables. Standard coefficients depict that all the observed indicators have values around 0.5 and are strongly related to their associated constructs [38]. Regarding direct and indirect effects, subsequent explanations are made.

The SM results showed that two constructs—environmental knowledge and defensive behavior—positively affect the household health status. Environmental knowledge positively influences the health status (0.30) and defensive behavior (0.01) of households at 0.5 [37]. Low-carbon consumption and environmental behavior is linked with environmental knowledge [45,46]. Individuals with a dearth of knowledge are more likely to harm the environment. Household’s defensive behavior has a direct positive effect on health status (0.14) and our hypothesis is confirmed. Hence, the findings show that households who are well aware of health and environmental risks are more involved in defensive practices.

The standardized coefficient of environmental quality on defensive behavior and household health status is statistically significant and has a negative impact. Environmental quality has a direct impact on health status [46] and an indirect impact on the defensive practices of households. This implies that the households who are putting efforts to adopt a green environment are less intent on adopting defensive behavior and vice versa. The most important factor related to health risk is waste disposal, which negatively affected health status and does not support our hypothesis. The findings indicate that inadequate waste management has serious effects on household health and results are consistent with the existing literature [14,23,24,25,26,27,28,29,30]. Moreover, waste disposal has a positive indirect impact on household defensive behavior, indicating an increase in improper waste disposal, leading to improved household defensive practices.

Estimated results are shown in Table 3. The standardized path coefficients of the households’ environmental knowledge and defensive behavior are 0.202 (*p* < 0.01) and 0.094 (*p* < 0.01) respectively. The impact of environmental knowledge and defensive practices on health is statistically significant at 1% confidence level. Results shows that direct effects of environmental knowledge and defensive practices on health are supported to our hypothesis. Our results are consistent with existing studies. Moreover, the environmental knowledge has largest effect (0.202) on household’s health status accompanied by defensive behavior.

While the impact of environmental quality is statistically significant −0.049 (*p* < 0.01), results shows that environmental quality has detrimental effects on household health status. The standardized path coefficients of waste disposal is statistically significant −0.273 (*p* < 0.01). Water, air, food and rats dwelling pollution through flies’ sources of several diseases in humans as plague, salmonellosis, trichinosis, endemic typhus dysentery, diarrhea and amoebic dysentery [46,47,48,49,50].

## 6. Conclusions and Policy Implications

We estimated an SM to test the hypotheses after we obtained a valid MM. Table 3 presents the results for the SM. The regression coefficient of waste disposal and environmental quality on health was negative and significant, suggesting the rejection of hypotheses H1, and H2. Waste disposal has a positive indirect effect on the defensive behavior of households, suggesting that a rise in excessive waste disposal leads to shift in defensive behavior, and environmental quality has a direct effect on health and an indirect impact on household standard precautions. The positive and significant regression coefficient of defensive behavior and environmental knowledge on health supports hypotheses H3 and H4.

The results of this study offer useful perspectives for policymakers. In the present case study, this could be related to the government’s solid waste management strategy. Government agencies and non-governmental organizations (NGOs) could participate to encourage households to segregate of waste at first source and propagate the benefits of a healthy environment. While environmental knowledge is an important factor regarding waste segregation and disposal it is recommended that government agencies and other associations tackle solid waste management by providing detailed information regarding different scenarios of waste disposal and segregation, and different households recycling forecasts at local and national levels. They should also provide details about the dangerous effects of illegal solid waste disposal on safety and the environment. In other words, the focus should be on shaping a proper system for collecting and disposing of waste. Accuracy and timelines of information are therefore important.

## Figures and Tables

**Table 1:** Statements and scales used for the four constructs.

Code	Description
HS1	Waste affect the mental well-being of the residents.
HS2	I am aware of the possible link between disease symptoms and improper waste disposal.
HS3	Has anyone of you suffer from the following waste-related diseases.
HS4	How would you evaluate your overall health status.
WD1	I always put garbage in a closed bin.
WD2	I always place plastic bag in bin.
WD3	I do segregate waste sometimes.
WD4	I positively engage in waste separation.
WD5	I adopted segregation behavior to minimize the waste management cost.
WD6	I feel responsible for segregating waste.
EQ1	I don’t notice any negative environmental changes in my vicinity.
EQ2	I don’t notice dumpsites near by me as the breading site for disease carrying vector?
EQ3	I don’t experience improper waste blocking.
EQ4	Water is not contaminated in my vicinity.
EQ5	I believe air is not polluted in my vicinity.
Ek1	I know how to segregate household waste properly.
EK2	Segregation of waste can help to enhance landfill life.
EK3	Waste disposal sites are not acting as breeding sites for disease carrying victors.
EK4	Household waste separation can help to decrease the morbidity rate.
EK5	Household waste separation can minimize the environmental damages.
EK6	I believe, control dumping can minimize greenhouse gas emissions.
DB1	I believe preventive measures should be taken to control mosquitoes.
DB2	I believe preventive measures should be used to control other insects.
DB3	I believe we should keep my drain free from blockage.
DB4	I believe we should adopt waste minimization practices at the first place.
DB5	I believe waste segregation can bring economic benefits.
DB6	I believe waste segregation practices can improve environmental quality in my vicinity.

**Table 2:** Reliability and validity test.

Goodness of Fit Measures	Recommendation Value	Structural Model (Results)
χ^2^	>3.00	3.713
CFI	>0.90	0.948
NFI	>0.90	0.931
RMSEA	<0.08	0.057

χ^2^ test statistics/df; CFI (comparative fit index); NFI (normed fit index); RMSEA (root mean square error of approximation).

**Table 3:** Results of the structural model (SM).

Structural Relations	Standardized Path Coefficient	S.E.	Hypothesis	Result
DA→HS	0.094 ***	0.024	H_1_	Supported
WD→HS	−0.273 ***	0.028	H_2_	Not supported
EK→HS	0.202 ***	0.024	H_3_	Supported
EQ→HS	−0.049 **	0.019	H_4_	Not Supported

Note: ***, **, significant at, 1%, and 5%.

## Data Availability

Data published in this study are available on request from the corresponding author. The data are not publicly available due to the policy of the research project.

## Appendix A

The data distribution of households for each socio-economic and demographic characteristic are presented in Table A1. Demographic statements that were incorporated in the survey included gender, age, education and income. A majority of (64.8%) of respondents in the sample are males and 35.2% are females. A substantial portion (37%) of households belongs to the early middle age group (21–30). The education level of households was low as follows: 23.9% of households were illiterate, 6.7% of households attended secondary school, 25.7% went to high school and just 21.7% of households had entered university. Regarding income, 21% of households claimed their monthly family income was less than 30.000 thousand rupees and 24% of respondents reported to being in the high income group.

**Table A1:** Social-economic information of respondents.

**Gender**	**Age**				
	15–20	21–30	31–40	41–50	>51
Female	153	221	119	47	11
Male	92	94	109	3	1
**Education**	**Income**				
	<30k	31k–50k	51k–70k	51k–70k	71k–100,000
Illiterate	92	57	21	7	26
Primary	30	10	9	3	5
Secondary	70	41	38	13	46
Higher	29	38	46	19	56
Professional	27	32	28	24	73

Source: [14].

**Table A2:** Correlations of the constructs.

	Health Status	Waste Disposal	Environmental Quality	Environmental Knowledge	Defensive Behavior
Health status	1				
Waste disposal	−0.111 ** (0.001)	1			
Environmental quality	−0.114 ** (0.001)	−0.15 (0.666)	1		
Environmental knowledge	0.294 ** (0.00)	−0.61 (0.76)	−0.71 * (0.038)	1	
Defensive behavior	0.69 * (0.044)	0.0179 ** (0.00)	−0.283 ** (0.00)	0.010 (0.769)	1

Note: *, **, significant at 1% and 5% and squared correlations in parentheses.
